# Nuclear T-STAR Protein Expression Correlates with HER2 Status, Hormone Receptor Negativity and Prolonged Recurrence Free Survival in Primary Breast Cancer and Decreased Cancer Cell Growth *In Vitro*

**DOI:** 10.1371/journal.pone.0070596

**Published:** 2013-07-29

**Authors:** Sandra Sernbo, Carl A. K. Borrebaeck, Mathias Uhlén, Karin Jirström, Sara Ek

**Affiliations:** 1 Department of Immunotechnology, CREATE Health, Lund University, Lund, Sweden; 2 Department of Biotechnology, AlbaNova University Center, Royal Institute of Technology, Stockholm, Sweden; 3 Department of Clinical Sciences, Division of Pathology, Lund University, Skåne University Hospital, Lund, Sweden; II Università di Napoli, Italy

## Abstract

T-STAR (testis-signal transduction and activation of RNA) is an RNA binding protein, containing an SH3-binding domain and thus potentially playing a role in integration of cell signaling and RNA metabolism. The specific function of T-STAR is unknown and its implication in cancer is poorly characterized. Expression of T-STAR has been reported in human testis, muscle and brain tissues, and is associated with a growth-inhibitory role in immortalized fibroblasts. The aim of this paper was to investigate the functional role of T-STAR through (i) survival analysis of patients with primary invasive breast cancer and (ii) experimental evaluation of the effect of T-STAR on breast cancer cell growth. T-STAR protein expression was analysed by immunohistochemistry (IHC) in tissue microarrays with tumors from 289 patients with primary invasive breast cancer, and correlations to clinicopathological characteristics, recurrence-free and overall survival (RFS and OS) and established tumor markers such as HER2 and ER status were evaluated. In addition, the function of T-STAR was investigated using siRNA-mediated knock-down and overexpression of the gene in six breast cancer cell lines. Of the tumors analysed, 86% showed nuclear T-STAR expression, which was significantly associated with an improved RFS and strongly associated with positive HER2 status and negative hormone receptor status. Furthermore, experimental data showed that overexpression of T-STAR decreased cellular growth while knock-down increased it, as shown both by thymidine incorporation and metabolic activity. In summary, we demonstrate that T-STAR protein expression correlates with an improved RFS in primary breast cancer. This is supported by functional data, indicating that T-STAR regulation is of importance both for breast cancer biology and clinical outcome but future studies are needed to determine a potential role in patient stratification.

## Introduction

Breast cancer is the most common cancer among women with 1.6 million new cases every year worldwide, and it is also the type of cancer with the highest mortality, causing more than 400 000 deaths annually [1]. However, the clinical behavior is diverse and stratification is needed to subgroup patients that benefit from different treatment strategies, including HER2 targeted treatment [2]. Today, prognostication is based on clinical parameters such as lymph node status, tumor size, age and histological grade; complemented by estrogen receptor (ER), progesterone receptor (PgR) and epidermal growth factor receptor (EGFR/HER2) status [3]–[5], which combined separate subgroups with different clinical behavior, including Luminal A, B, HER2 and basal-like tumors [6], [7]. However, it is clear that also within subgroups, such as HER2 positive tumors, patients respond differently to selected therapy [8] and that further biological insight is needed. A major bottleneck in translational research has been the lack of validated antibodies to study novel potentially clinical relevant antigens.

We have previously developed antibodies targeting tumor-associated antigens and screened them for differential binding to tumor and normal cells by immunohistochemistry (IHC) [9]. One of the antigens identified as being able to separate normal from malignant cells was the RNA-binding protein T-STAR (testis-signal transduction and activation of RNA).

RNA binding proteins are of major importance as they impact every process in the cell; they may act as splicing and polyadenylation factors, transport and localization factors, stabilizers and destabilizers, modifiers and chaperones [10]. T-STAR is a relatively uncharacterized RNA binding protein belonging to the STAR family, and has important cellular functions such as RNA processing, signal transduction and cell cycle regulation [11], [12]. All members share a STAR domain, which is required for RNA-binding and the ability to be modified by several post-translational mechanisms such as phosphorylation and methylation, which affect the RNA binding capacity [13]–[17]. A unique feature of these proteins is their capacity to integrate external and internal cell signaling directly to changes in transcription and processing of target RNAs, as they contain both proline rich binding sites for SH3 domains, often found in proteins involved in cell signaling, as well as a RNA binding KH domain [18]. This rapid way of signal transduction has an important role in RNA metabolism [13], [16]. T-STAR belongs to the same subgroup as Sam68 and SLM-1, showing 65–70% sequence identity in the STAR domain [16]. Sam68 is by far the most studied member in the STAR family and is more ubiquitously expressed than T-STAR, which is restricted to healthy testis, muscle and brain [17]. Of major interest, T-STAR has been suggested to mediate growth arrest in chicken embryo fibroblasts [18] and to regulate telomerase activity in human colon cancer cell lines [19], but its protein expression in primary tumors has not been assessed to date, and possibilities have been limited by lack of validated antibodies targeting T-STAR in IHC.

In this study, we provide the first detailed investigation of the role of T-STAR in breast tumors, using IHC on a cohort of 289 cases of invasive breast cancer together with functional investigation on the impact of forced decrease and increase on expression levels in breast cancer cell lines. Of major importance, we show that the expression of T-STAR significantly correlates with improved recurrence free survival (RFS) in agreement with our functional data showing that T-STAR induces decreased cancer cell growth rates *in vitro*.

## Methods

### Ethics Statement

All EU and national regulations and requirements for handling human samples (se list below) have been fully complied with during the conduct of this project.

1. Decision no. 1110/94/EC of the European Parliament and of the Council (OJL126 18,5,94).
2. The Helsinki Declaration on ethical principles for medical research involving human subjects, i.e. Declaration of Helsinki - Ethical Principles for Medical Research Involving Human Subjects (2000).
3. EU Council Convention on human rights and Biomedicine, i.e. The Council of Europe’s Convention for the Protection of Human Rights and Dignity of the Human Being with Regard to the Application of Biology and Medicine: Convention on Human Rights and Biomedicine.

Furthermore, we have an ethical approval (Dnr 445/07) from the Malmo/Lund regional ethical committee for the collection of tissue samples used in the project, which include an informed oral consent from all patients included in the study, as documented in each patient journal. Patients were informed orally and opting out was an option. Written consent was not obtained because the Malmo/Lund regional committee decided that this was not necessary. The opting out method was approved by the Malmo/Lund regional committee.

### Patients

IHC analysis was performed on tissue microarrays (TMA:s) with tumor specimens from an unselected cohort originally consisting of 512 cases of invasive breast cancer diagnosed at the Department of Pathology, Malmö University Hospital, between 1988–1992. IHC evaluation of T-STAR expression was performed on 289 cases. Median age at diagnosis was 66 years (27–96 years). Histopathological, clinical and treatment data were obtained from the clinical- and/or pathology records. Information on vital status and cause of death was obtained from the Swedish Cause of Death Registry. Of the 289 patients fourteen had received chemotherapy, and 102 had received endocrine therapy (tamoxifen). For 62 of the patients, information on adjuvant treatment was lacking. The clinicopathological characteristics for the cohort have been described elsewhere [20] and can also be found in Supporting information (Table S1).

### TMA Constructions

Along with the histological re-evaluation, areas representative of invasive tumor were marked on haematoxylin & eosin stained sections. Two 0.6 mm tissue cores were then taken from the corresponding paraffin block and mounted in triplicates in recipient blocks. One set of TMAs were constructed using a manual device (MTA-1) and one using an automated device (ATA-27, Beecher Instruments, WI, USA).

### Immunohistochemistry

IHC were assessed on four µm sections that were dried, deparaffinized, rehydrated and microwave treated as previously described [21]. The T-STAR antibody (HPA000500) was developed as previously described and validated using IHC on different tissues within the Human Protein Atlas (HPA)-project [9] (Atlas Antibodies, Stockholm, Sweden). The antibody recognizes the sequence.

GEGKDEEKYIDVVINKNMKLGQKVLIPVKQFPKFNFVGKLLGPRGNSLKRLQEETLTKMSILGKGSMRDKAKEEELRKSGEAKYFHLNDDLHVLIEVFAPPAEAYARM. ER status had previously been determined on triplicate, manually constructed, sections (6×0.6 mm cores) and PgR status on single, automatically constructed, sections (2×0.6 mm cores), using the Ventana Benchmark system (Ventana Medical Systems Inc., AZ, USA) with prediluted antibodies (Anti-ER Clone 6F11 and anti PgR Clone 16). In line with the current clinical guidelines, 10% nuclear positivity was used as cut-off for both hormone receptors. HER2 status had been assessed on duplicate cores using the Ventana Benchmark system with a pre-diluted antibody (Pathway CB-11, 760–2694). Staining was evaluated semi-quantitatively according to a standard protocol (HercepTest).

Apart from the clinically established markers, a number of investigative markers have been analyzed in this cohort, i.e., VEGF-A, VEGFR2 and cyclin D1 as described elsewhere [22]–[24].

### Statistical Analysis

Spearmans ρ and χ^2^ linear by linear association was used for comparison between T-STAR expression and other parameters. RFS and OS were estimated according to the Kaplan-Meier method and the log-rank test to compare survival between strata. A Cox multivariate proportional model was used to investigate the effect on RFS in relation to established clinicopathological parameters.

### Cultivation of Breast Cancer Cell Lines

Six breast cancer cell lines were used as *in vitro* models; MDA-MB-231, PMC42, JIMT-1, SK-BR3, T47D and L56Brc1. The cell lines were either purchased from ATCC or generously donated (for more information see Table 1) [25]–[28]. All cell lines, except L56Brc1, were cultured in RPMI-1640 medium (HyClone, South Logan, UT) supplemented with 10% (v/v) fetal calf serum and 2 mM L-Glutamine (both Invitrogen, Carlsbad, CA, USA), hereafter referred to as R10 medium. L56Brc1 was cultured in R10 supplemented with 0.01 mg/ml human recombinant insulin (Sigma-Aldrich, St. Louis, MO, USA).

**Table 1:** Specification of breast cancer cell lines used in the experiments.

Cell line	Tumor type	Source	ER	T-STAR
**SK-BR-3** 3	adenocarcinoma	pleural effusion metastasis	−	+ low
**MDA-MB-231** 1	adenocarcinoma	pleural effusion	−	+ high
**L56Br-C1** 1		lymph node metastasis	−	+ high
**JIMT1** 1	ductal carcinoma	pleural metastasis	−	+ low
**PMC42** 2	Stem-cell like	pleural effusion	+	+ high
**T47D** 3	ductal carcinoma	pleural effusion	+	+ low

1 Kindly provided by Cecilia Hegardt, Department of Oncology, Clinical Sciences, Lund University, Skåne University Hospital.

2 Kindly provided by Paolo Cifani, Department of Immunotechnology, Lund.

3 ATCC.

### Knock-down and Overexpression of T-STAR using Lipofectamine

Cells were knocked-down with siRNA targeting T-STAR (T3: sense 5′→3′: GGAUGAAGAAAAGUACAUCtt, antisense 5′→3′: GAUGUACUUUUCUUCAUCCtt) (Ambion, Austin, TX, USA) using the Lipofectamine™ 2000 protocol (Invitrogen). A scrambled sequence (scr), a wildtype (wt) control (transfected in absence of siRNA) and a pmaxGFP™-vector (to evaluate transfection efficiency) were used as controls.

T-STAR was overexpressed using an OmicsLink™Expression Clone for T-STAR (EX-P0058-M46) and a GFP control vector (EX-EGFP-M02) was used as reference (both from GeneCopoeia, Germantown, MD, USA). Also, wt cells were used as a control (transfected without plasmid). In the transfection, 1 µg of plasmid was mixed with Lipofectamine 2000 and added to the cells. Cells were harvested (24 and 48 hours after transfection) and samples were collected for Real-Time-qPCR (RT-qPCR), Western blot and proliferation assays.

### RNA Isolation and RT-qPCR

The relative quantity (RQ) of T-STAR in the different samples was determined by RT-qPCR. Cells were lysed and cDNA prepared using the iScript™ RT-qPCR Sample Preparation and iScript cDNA Synthesis kit reagents (BIO-RAD, Hercules, CA, USA). Briefly, 2.5*10^4^ cells were mixed with 50 µl of lysis solution, vortexed for 30 seconds, centrifuged for 2 min at 15 000 g and the supernatant was subsequently collected. After reverse transcription using 2 µl of lysate, 3µl cDNA was used in the RT-qPCR reaction (using the SsoFast EvaGreen Supermix with low ROX, BIO-RAD) with the following primers specific either for *T-STAR* or the house-keeping gene *GAPDH* (500 nM, Eurofind MWG Operon, Ebersberg, Germany); *GAPDH*: 5′-TGGTATCGTGGAAGGACTC-3′ and 5′-AGTAGAGGCAGGGATGATG-3′, *T-STAR*: 5′-GGGACATGCTTTGGAAGAAA-3′ and 5′-CTTGTACGCAAGGTGGGTTT-3′. The RQ was calculated as 2^−(ΔΔCT (T-STAR-GAPDH))^. The RQ value for the scrambled control was set to 1 for each cell line and the SD was calculated with a 95% confidence interval.

### Protein Extraction, Western Blot and Quantification of T-STAR Expression

Protein was extracted and quantified as previously described [29]. Briefly, cells were harvested, washed and placed in 100 µl lysis-buffer (1% Ipegal/Protease Inhibitor cocktail (Roche, Basel, Switzerland) in PBS). The protein lysate (15–25 µg ) was then run on a NuPAGE 10% Bis-Tris gel (Invitrogen) under reducing conditions for 50 min at 135 V. Proteins in the gel were blotted on PVDF iBlot Transfer stacks in the iBlot gel transfer device (both Invitrogen) and blocked in 5% milk/PBS O/N. The T-STAR protein was detected using a T-STAR antibody (Santa Cruz) and a secondary HRP-labeled antibody (DAKO, Glostrup, Denmark). Signals were visualized with the SuperSignal West Femto Max Sensitivity Substrate (Pierce Biotechnology Inc., Rockford, IL) and analyzed in the Image Studio software program (Logitech, Freemont, CA, USA). The protein bands were quantified with the Quantity One software (BIO-RAD) using the volume tool and global background subtraction. The volume of the bands was compared to the scrambled siRNA (knock-down experiments) or to the wt (overexpression experiments) control, which were both set to 1 (100%).

### Proliferation Assay and Cell Cycle Experiments

The proliferation assays were performed using [methyl-3H]-thymidine incorporation as previously described [30]. Briefly, cells were seeded in 96-well plates and cultivated for 48 or 72 h with addition of Methyl-3H-thymidine during the last 8 h. Cells were harvested using a Tomtec harvester 96 (Tomtec, Hamden, CT, USA) and scintillation measured in a 1450 Microbeta liquid scintillation counter (PerkinElmer, Waltham, MA, USA). Proliferation was also evaluated with the WST-1 cell proliferation reagent (Roche), where cells were seeded and cultivated as above. The WST-1 substance is cleaved by mitochondrial dehydrogenases with a rate corresponding to the number of viable cells in the culture. During the last 2 h, 10 µl of the WST-1 reagent was added to each well and absorbance of the cleaved product was measured in a spectrophotometer as A_450 nm_ –A_690 nm_. In the statistical analysis, ±1 SD was used for both proliferations assays. In general, data shown is representative of three individual experiments. Furthermore, a two-tailed paired t-test (in Excel assuming non equal variance) was performed on all data, and significance defined as a p-value below 0.05. Cell cycle analysis was performed according to standard procedure, as described in detail previously [31].

## Results and Discussion

**Figure 1:**
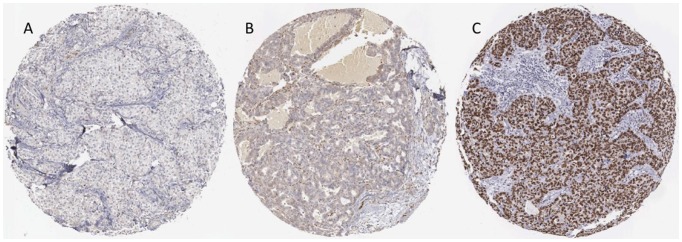
IHC analysis of T-STAR on primary invasive breast cancer where A) shows a negative case, B) a case with cytoplasmic staining and C) a case with nuclear T-STAR staining.

In this study, we have explored the prognostic and functional properties of the RNA-binding protein T-STAR. Using IHC, T-STAR was found to be expressed in various fractions and intensities in the tumor cell nuclei while cytoplasmic staining, when present, was generally observed in all cells with varying intensity. The fraction of positive nuclei was scored and samples divided into groups as follows, 0 =  <10%, 1 = 11–50%, 2 = 51–75% and 3 =  >75%. The nuclear and cytoplasmic staining intensity was scored as 0 =  absent, 1 =  weak, 2 =  moderate and 3 =  strong and 0 =  absent, 1 =  weak and 2 =  moderate, respectively. Examples of tumors with negative and strong staining are shown in Figure 1. No membranous staining was observed and nuclear and cytoplasmic staining correlated strongly (p<0.001). The dual localizations are in agreement with previous studies where the STAR family member QKI-5 has been found to be shuttled between the two compartments [32] and also Sam68 is cytoplasmically expressed in various cancerous tissues [33]–[35]. In this study, T-STAR expression was similar in the *in situ-* and invasive components in cases where both entities had been sampled. Also in normal glands and ducts, scattered nuclear and sometimes cytoplasmic positivity was seen, seldom exceeding 50% of the cells.

Furthermore, correlation between T-STAR and established clinicopathological parameters and tumor markers was investigated and the results are presented in Table 2. Nuclear staining was defined by intensity, but similar associations were seen when the fraction of positive cells was assessed (data not shown). Interestingly, nuclear sub-localization of T-STAR was strongly associated with positive HER2 status and inversely associated with hormone receptor positivity. There was also an inverse correlation between T-STAR expression and age at diagnosis. No significant correlations to other clinicopathological parameters were observed. T-STAR expression was also correlated to investigative markers, with both nuclear and cytoplasmic staining being significantly associated with VEGF expression (p = 0.001 and 0.002 respectively) and cytoplasmic, but not nuclear, staining being significantly associated with expression of VEGFR2 (p = 0.004). In line with its inverse relationship to hormone receptor status, nuclear but not cytoplasmic T-STAR expression was inversely associated with cyclin D1 expression (p = 0.001). Of note, there was no significant association with Ki-67. Ki-67 is considered an important prognostic marker for invasive breast cancer [36] but is mainly used in combination with ER and PR as recommended by the 2011 St. Gallen Consensus Conference summary [37].

**Table 2:** T-STAR nuclear and cytoplasmic expression in relation to patient- and tumor characteristics in the total cohort (χ2 test for linear trend).

	T-STAR nuclear intensity n (%)					T-STAR cytoplasmic intensity n (%)			
	0	1	2	3	*p-value*	0	1	2	*p-value*
**All**	44 (15)	130 (45)	83 (30)	32 (11)		52 (18)	172 (60)	65 (22)	
**Menopausal status** (unknown = 83)									
pre-	5 (11)	11 (8)	11 (13)	6 (19)		7 (13)	18 (10)	8 (12)	
post-	31 (70)	92 (71)	36 (43)	14 (44)	0.01	34 (65)	108 (63)	30 (46)	0.65
**Tumor size** (unknown = 4)									
<20 mm	24 (55)	84 (65)	48 (58)	12 (38)		25 (48)	109 (63)	34 (52)	
> = 20 mm	20 (45)	45 (35)	34 (41)	18 (56)	0.17	27 (52)	60 (35)	30 (46)	0.72
**Nodal status** (unknown = 30)									
neg	26 (59)	70 (54)	41 (49)	18 (56)		27 (52)	90 (52)	38 (58)	
pos	17 (39)	44 (34)	31 (37)	10 (31)	0.97	20 (38)	61 (35)	21 (32)	0.46
**NHG** (unknown = 5)									
I	7 (16)	40 (31)	14 (17)	7 (22)		8 (15)	44 (26)	16 (25)	
II	13 (30)	54 (42)	37 (45)	9 (28)		18 (35)	74 (43)	21 (32)	
III	24 (55)	34 (26)	31 (37)	14 (43)	0.86	26 (50)	50 (29)	27 (42)	0.35
**ER Status** (unknown = 7)									
Negative	2 (5)	6 (5)	13 (16)	11 (34)		6 (12)	13 (8)	13 (20)	
Positive	41 (93)	121 (93)	69 (83)	19 (59)	<0.001**	45 (87)	155 (90)	50 (77)	0.096
**PgR Status** (unknown = 54)									
Negative	13 (30)	28 (22)	27 (33)	19 (59)		17 (33)	43 (25)	27 (42)	
Positive	19 (43)	77 (59)	42 (51)	10 (31)	0.002**	25 (48)	91 (53)	32 (49)	0.44
**Age** (unknown = 4)									
<50 years	4 (9)	11 (8)	20 (24)	6 (19)		10 (19)	19 (11)	12 (18)	
>50 years	40 (91)	118 (91)	62 (75)	24 (75)	0.007**	42 (81)	150 (87)	52 (80)	0.94
**Ki-67** (unknown = 13)									
0–10%	14 (32)	63 (48)	24 (29)	7 (22)		14 (27)	70 (41)	24 (37)	
11–25%	14 (32)	37 (28)	28 (34)	11 (34)		22 (42)	56 (33)	12 (18)	
>25%	14 (32)	26 (20)	28 (34)	11 (34)	0.059	14 (27)	39 (23)	26 (40)	0.71
**HER2 status** (unknown = 90)									
0	17 (39)	50 (38)	25 (30)	6 (19)		18 (35)	64 (37)	16 (25)	
1	11 (25)	27 (21)	25 (30)	9 (28)		12 (23)	40 (23)	20 (31)	
2	3 (7)	6 (5)	7 (8)	2 (6)		3 (6)	9 (5)	6 (9)	
3	2 (5)	1 (1)	4 (5)	4 (13	0.007**	4 (8)	5 (3)	2 (3)	0.69

**Figure 2:**
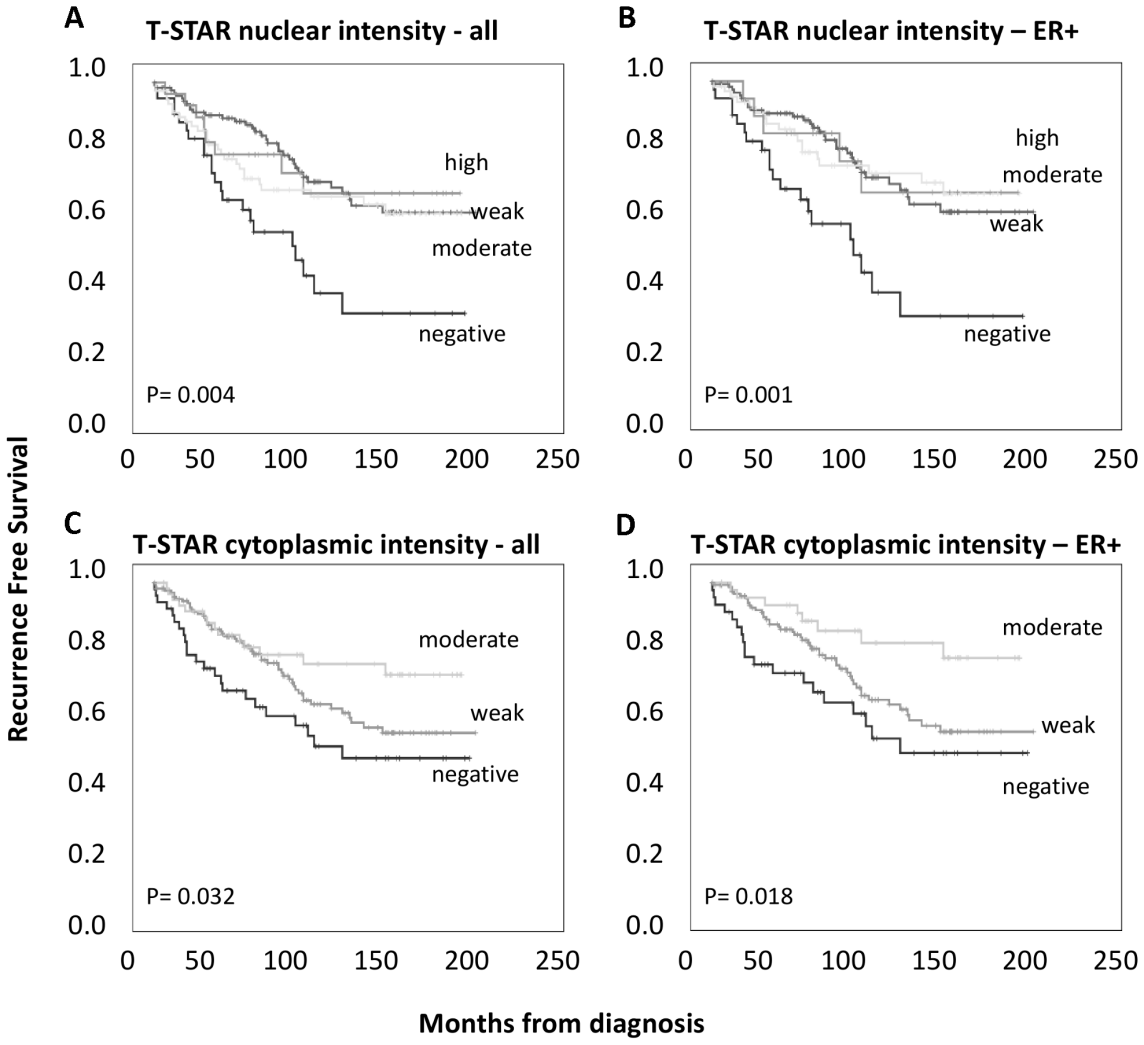
Kaplan-Meier curves showing T-STAR expression correlated to survival. A) Nuclear T-STAR expression in all cases. B) Nuclear T-STAR expression in ER+ cases only. C) Cytoplasmic T-STAR expression in all cases. D) Cytoplasmic T-STAR expression in ER+ cases only.

**Figure 3:**
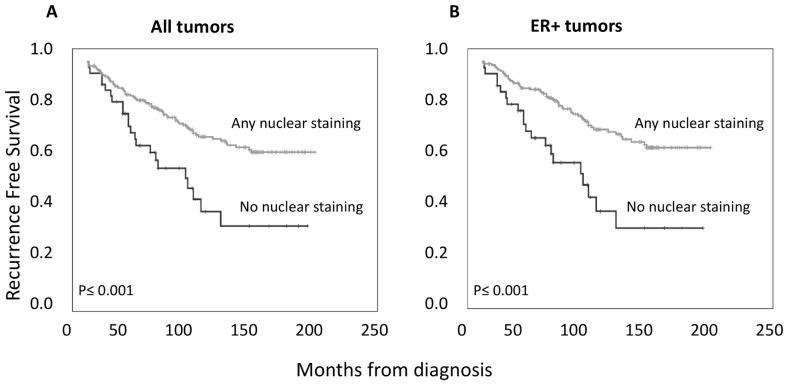
Kaplan-Meier curves correlating T-STAR expression to survival using a dichotomized variable where any nuclear staining and intensity have been grouped together. A) Showing T-STAR expression in all cases. B) Showing T-STAR expression in ER+ cases only.

In addition, as shown in Figure 2, both nuclear and cytoplasmic T-STAR expressions were correlated to RFS in all and ER positive tumors, while no such correlation was found in ER negative tumors (data not shown). Nuclear expression was significantly associated with improved RFS for all intensities and the trend was almost identical for the nuclear fraction (data not shown). Absent cytoplasmic staining was also associated with a reduced RFS, but there was a difference between weak and moderate staining as illustrated in Figure 2 C-D. A dichotomized variable using a combination of nuclear fraction and intensity with 0 =  no staining and 1 =  any nuclear fraction or intensity is shown in Figure 3. Absent versus present nuclear T-STAR expression remained significant in a multivariate Cox regression model, adjusted for prognostic clinicopathological parameters, as shown in Table 3. Cytoplasmic staining (0 vs 1–2) was significant in the univariate but not multivariate model (data not shown). Neither nuclear nor cytoplasmic T-STAR sub-localization was associated with overall or breast cancer specific survival (data not shown). The correlation between T-STAR expression and RFS indicates that T-STAR has potential as a prognostic marker, but further confirmatory studies are warranted. Even though the prognostic power of T-STAR was independent of adjuvant therapy in this study (Table 3), its potential utility as a predictive marker should also be evaluated in future studies, preferably in a randomized setting.

**Table 3:** Cox uni- and multivariate analysis of Recurrence Free Survival according to nuclear T-STAR expression.

Covariate category	Univariate			Multivariate		
	HR	95% CI	*p-value*	HR	95% CI	*p-value*
**Tumor size**						
<20 mm	1.0			1.0		
≥20 mm	2.1	(1.5–2.9)	<0.001**	1.2	(0.73–2.0)	*0.45*
**Nodal status**						
neg	1.0			1.0		
pos	3.3	(2.4–4.7)	<0.001**	3.4	(1.7–6.0)	<0.001**
** NHG**						
I–II	1.0			1.0		
III	3.2	(2.3–4.4)	<0.001**	2.1	(1.3–3.6)	0.003**
**ER Status**						
Positive	1.0			1.0		
Negative	1.7	(1.1–2.6)	0.017**	1.3	(0.64–2.0)	*0.41*
**Age**						
>50 years	1.0			1.0		
<50 years	1.4	(0.92–2.0)	*0.13*	1.1	(0.56–2.0)	*0.87*
**HER2 status**						
3	1.0			1.0		
0–2	0.98	(0.59–1.6)	*0.95*	0.98	(0.43–2.2)	*0.96*
**Adjuvant therapy**						
Treated (chemo/endocrine)	1.0			1.0		
Untreated	0.36	(0.25–0.53)	<0.001**	1.3	(0.59–3.0)	*0.49*
**T-STAR nuclear staining**						
negative	1.0			1.0		
positive	0.43	(0.26–0.70)	0.001**	0.48	(0.27–0.85)	0.011**

Note: T-STAR nuclear positive staining is defined as >10%.

**Figure 4:**
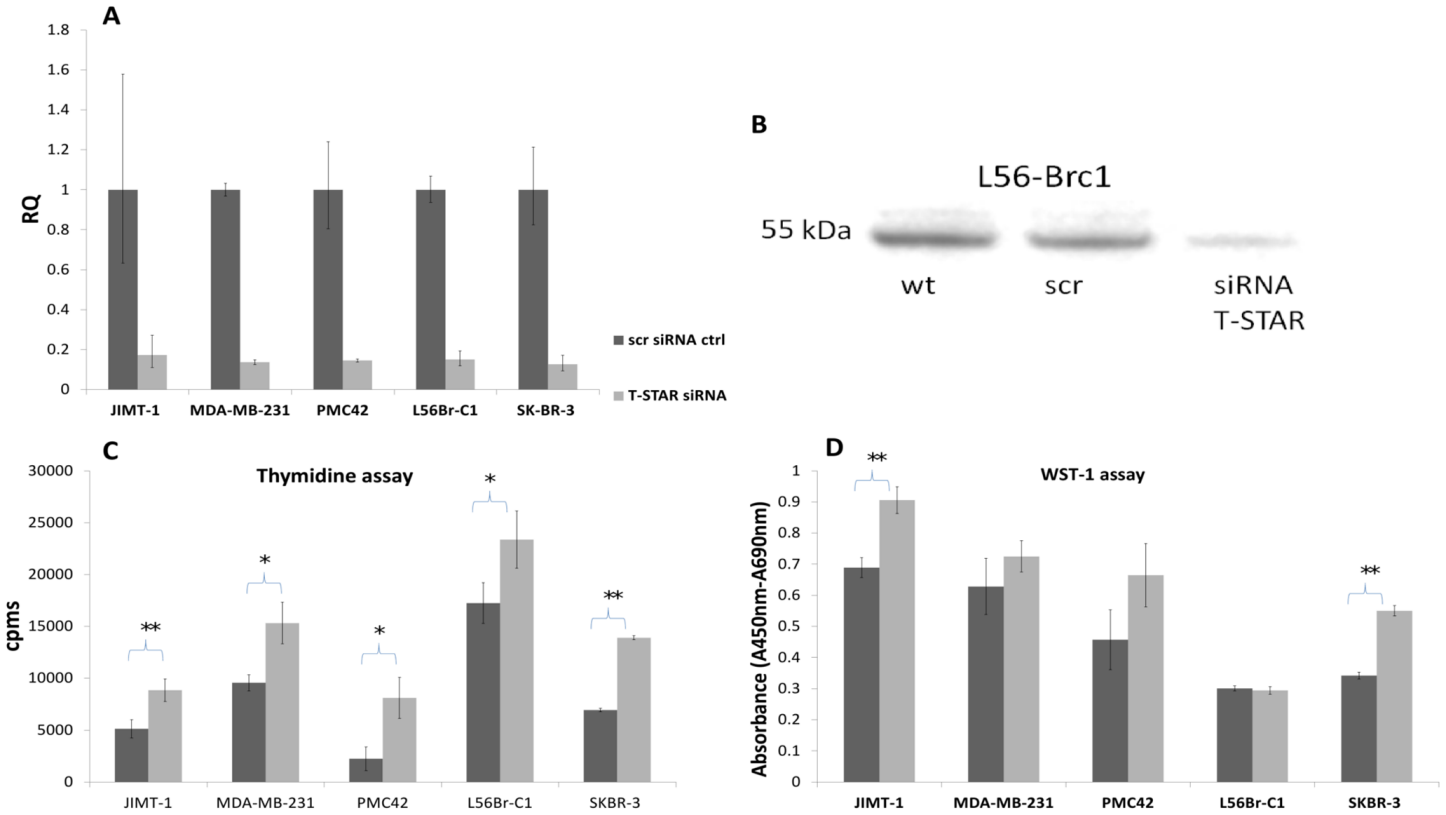
Increased proliferation after knock-down of T-STAR in five breast cancer cell lines. A) Reduction in T-STAR mRNA levels after knock-down as assessed by RT-qPCR. B) Representative data showing, reduction (17%) in T-STAR protein levels (WB) compared to wt (130%) and scrambled control cells (100%) in the L56Brc1cell line after knock-down. C) Increased proliferation at 48 h (JIMT-1, MDA-MB-231 and L56Br-C1) or 72 h (PMC42 and SKBR-3) after knock-down compared to the scrambled control using thymidine incorporation (measured as cpms (counts per minutes)). Of note, values for JIMT-1 are scaled by a factor 10 for the clarity of presentation. D) An increase in proliferation was also seen measured by the WST-1 assay after 48 h (JIMT-1 and MDA-MB-231) or 72 h (PMC42, L56Br-C1 and SKBR-3). Significance is marked by a * where the p-value ≤0.05 and ** when ≤0.01.

**Figure 5:**
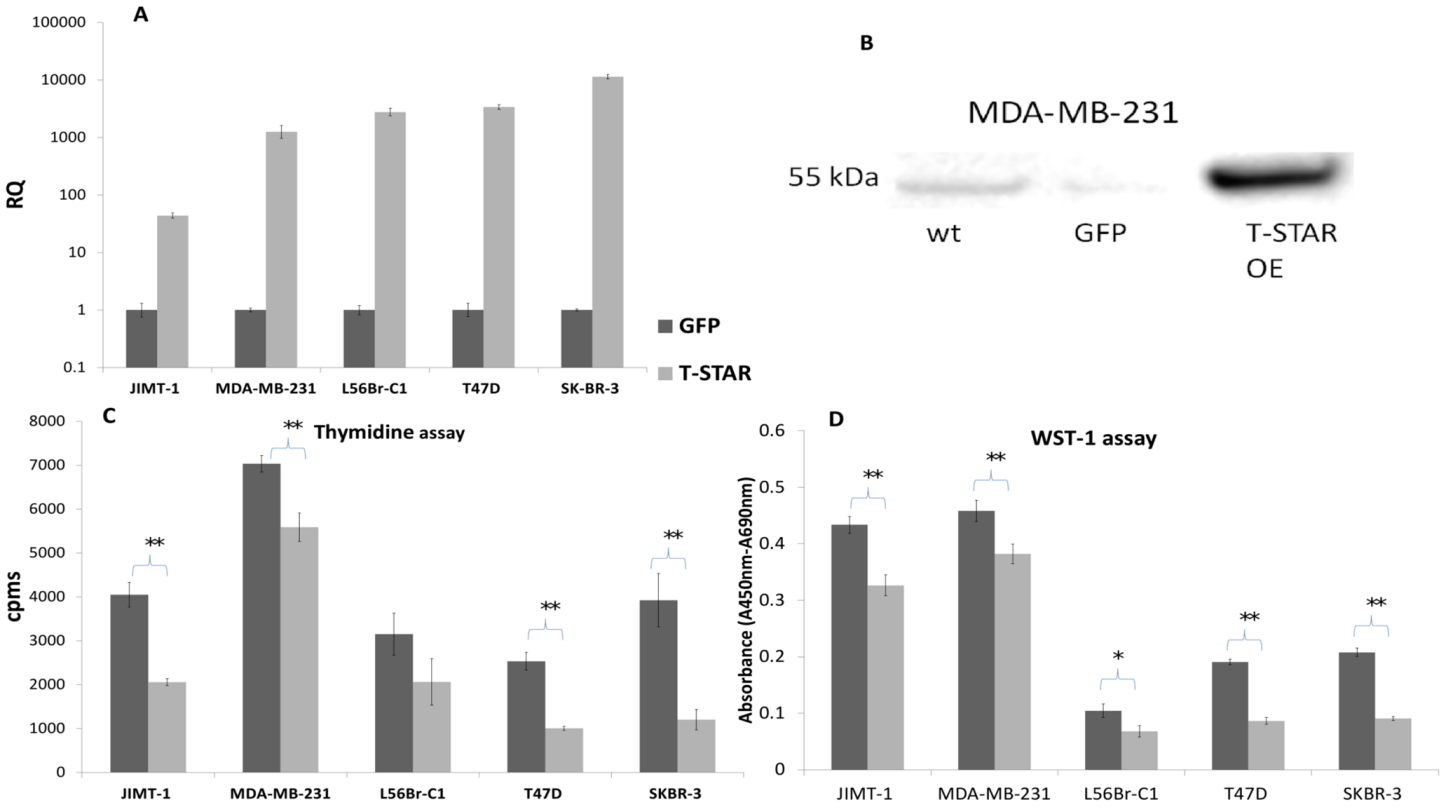
Decreased proliferation after overexpression of T-STAR in five breast cancer cell lines. A) Increased T-STAR mRNA levels after overexpression measured by RT-qPCR. B) Increased (676%) T-STAR protein level (WB) compared to GFP (16%) and wt (100%) control cells in the MDA-MB-231 cell line after overexpression. C) A decrease in proliferation could be detected at 48 h after overexpressing T-STAR using thymidine incorporation and D) after 48 h (PMC42, L56Br-C1 and T47D) or 72 h (JIMT-1 and MDA-MB-231) upon assessment of enzymatic activity (WST-1 assay). Significance is marked by a * where the p-value ≤0.05 and ** when ≤0.01.

The implications of T-STAR expression in cells are largely unknown, although previous studies have shown a growth arresting effect in immortalized fibroblast [18], [38]. In order to investigate the potential growth regulatory role in breast cancer, we knocked-down and overexpressed the gene in six human breast cancer cell lines. The siRNA and plasmid transfection rates were estimated using two different GFP control vectors (pmaxGFP™ and EX-EGFP-M02). The rates varied between 61–90% in the different cell lines using the pmaxGFP™-vector, while the EX-EGFP-M02-vector showed lower GFP expression (20–73%), which can be explained by the larger genome (8400 bp compared to 3486 bp). The lower transfection rate for L56Brc1 correlated to a lower effect on proliferation in the thymidine and WST-1 assays but still resulted in significant protein reduction. The knock-down and overexpression were confirmed both at the mRNA level and at the protein level (Figure 4A and B and 5A and B respectively). Of note, the successful reduction of signal in wb confirms the specificity of the developed antibody towards the T-STAR protein. Efficient knockdown (>80%, measured as RQ) was achieved in all five cell lines. A wt control was included in the siRNA experiments but yielded similar results as the scrambled control (data not shown). The large standard deviation for JIMT-1 is explained by the relatively low wild-type expression, close to detection limit. Efficient knock-down of T-STAR could also be confirmed at the protein level in several of the cell lines. In JIMT-1, which has a low mRNA expression of T-STAR, no protein was detected. Representative data showing a strong, >5 fold reduction (17%) in T-STAR protein level (detected at 55 kDa) compared to scrambled control cells (100%) for L56Brc1 cells is shown in Figure 4B.

The knock-down of T-STAR correlated to an increase in proliferation measured both by thymidine incorporation (Figure 4C) and WST-1 proliferation reagent (Figure 4D). The increase was significant (p<0.05) and generally of larger magnitude in the thymidine incorporation assay compared to the WST-1 assay, where only two cell lines reached significance (JIMT-1 and SK-BR-3). This indicates that the thymidine assay is more sensitive. However, the two methods are complementary as they assess proliferation differently, either as DNA replication (thymidine incorporation) [39] or enzymatic activity (WST-1 assay) [40]. For all cell lines but L56Brc1 the increase in proliferation ranged from 50% to 262% assessed through thymidine incorporation. Using assessment of enzymatic activity, the increase in proliferation ranged from 15% to 61%, excluding the L56Brc1cell line. Of note, JIMT-1 showed high cpms values in the thymidine assay and for the clarity of presentation; a scale factor of 10 has been used.

After overexpression, T-STAR mRNA increased 44 to 10 000 fold in JIMT-1 and SK-BR-3, respectively. A representative example of corresponding increase in protein level is shown in Figure 5B. In MDA-MB, T-STAR protein level increased 6.8 fold compared to the wt. The GFP control produced a weak band of only 16% compared to the wt.

Overexpression of T-STAR resulted in decreased (p<0.05) proliferation in both assays (Figure 5C and D), with L56Br-1 as an exception in the thymidine assay. The reduction in proliferation ranged from 21% in MDA-MB-231 to 69% in SK-BR-3 in the thymidine assay. In the WST-1 assay the reduction ranged from 17% to 57%. Generally, GFP control cells showed reduced proliferation compared to wt mock transfected cells emphasizing the importance of relevant control vectors. In combination, these results show a growth regulatory role of T-STAR upon increased and decreased gene expression, indicating a growth-inhibitory function *in vivo*. Furthermore, cell cycle analysis after overexpression of T-STAR showed a decreased fraction of cells in S phase compared to GFP control (31% compared to 38% for JIMT-1 at 24 h and 19% compared to 25% for MDA-MB-231 at 48 h) and an increased fraction of cells in G0/G1 (55% compared to 48% for JIMT-1 and 66% compared to 62% for MDA-MB-231 at 48 h). Of note, cells have poor viability after T-STAR overexpression and as only cells with intact morphology can be analysed, the differences are less pronounced compared to the proliferation data. However, data from both knock-down and overexpression studies are in agreement with the survival data presented here, where patients with expression of T-STAR showed an increased RFS. It is also supported by previous work where expression is associated with arrested cell growth [18], [38].

Further studies are needed to understand the molecular mechanism of T-STAR growth regulation. To get further insight into the function of T-STAR, previous studies on Sam68, one of its closest relatives, are of value. Sam68 is bound and phosphorylated by many different kinases, i.e. Src, PI3K and PLCγ1, and the protein seems to have many target mRNAs, among others CD44, Bcl-X, mTOR and cyclin D1 [16], [41]. In the TNF receptor pathway, Sam68 is required for both NF-κB activation and apoptosis signaling [42]. T-STAR, on the other hand, has only been found to interact with one kinase; the breast tumor kinase (BRK), and with only one SH3 binding domain it is not likely to serve as a scaffold protein [16], [43]. Interestingly, BRK is the only kinase that co-localizes with Sam68 in the nucleus [16], [44], suggesting that this kinase, which has been associated to breast cancer motility [44], is closely connected to the function of the RNA binding proteins. Thus, future studies of the relationship between T-STAR and BRK are of importance to elucidate the molecular function of T-STAR in breast cancer.

## Conclusions

Using a novel antibody reagent, IHC analysis revealed an association between the RNA-binding protein T-STAR and RFS of patients afflicted by primary invasive breast cancer. The expression of T-STAR also correlated with positive HER2 status and hormone receptor negativity. This finding is of major interest as it offers potential as a complement to the current biomarkers ER, PgR and HER2 in prognosis of the disease. In agreement with clinical data, functional studies in breast cancer cell lines showed a strong correlation between T-STAR expression and proliferation, indicating that T-STAR regulation is of importance for both clinical outcome and also breast cancer tumor growth.

## Supporting Information

Table S1 **Clinicopathological characteristics of the patients.**
(DOCX)
