# Supplementary material for: Nuclear T-STAR Protein Expression Correlates with HER2 Status, Hormone Receptor Negativity and Prolonged Recurrence Free Survival in Primary Breast Cancer and Decreased Cancer Cell Growth In Vitro
Source: PLoS One. 2013 Jul 29;8(7):e70596. doi: 10.1371/journal.pone.0070596 (PMC3726654; doi:10.1371/journal.pone.0070596)
Supplement: Table S1 — Clinicopathological characteristics of the patients. (DOCX) [file pone.0070596.s001.docx]

| *Table S1. Clinicopathological characteristics* | |
| --- | --- |
| *Histological subtype* | **Number of cases (%)** |
| Ductal carcinoma NOS | 199 (68.9%) |
| Lobular carcinomas | 41 (14.2%) |
| Mixed ductal-lobular carcinoma | 20 (6.9%) |
| Tubular carcinoma | 13 (4.5%) |
| Medullary carcinoma | 8 (2.8%) |
| Mucinous carcinomas | 8 (2.8%) |
|  |  |
| *Histological grade* |  |
| NHG I | 68 (23.5%) |
| NHG II | 115 (39.8%) |
| NHG III | 105 (36.3%) |
|  |  |
| *Recurrence after 207 months* |  |
| Yes | 92 (32.2%) |
| No | 194 (67.8%) |
|  |  |
| *Distribution recurrences* |  |
| local | 17 (6%) |
| regional | 5 (2%) |
| distant | 56 (20%) |
|  |  |
| *OS after 207 months* |  |
| Alive | 124 (42.9%) |
| Dead, unspecific cause | 108 (37.4%) |
| Dead, breast cancer | 56 (19.4%) |
|  |  |
| *Tamoxifen treatment* |  |
| Yes | 102 (35.3%) |
| No | 116 (40.1%) |
|  |  |
| *Chemotherapy treatment* |  |
| Yes | 14 (4.8%) |
| No | 206 (71.3%) |
